# Metabolic Biomarkers of Red Beetroot Juice Intake at Rest and after Physical Exercise

**DOI:** 10.3390/nu15092026

**Published:** 2023-04-22

**Authors:** Ottavia Giampaoli, Cristian Ieno, Fabio Sciubba, Mariangela Spagnoli, Alfredo Miccheli, Alberta Tomassini, Walter Aureli, Luigi Fattorini

**Affiliations:** 1 NMR-Based Metabolomics Laboratory (NMLab), Sapienza University of Rome, 00185 Rome, Italy; ottavia.giampaoli@uniroma1.it (O.G.); fabio.sciubba@uniroma1.it (F.S.); m.spagnoli@inail.it (M.S.); 2 Department of Environmental Biology, Sapienza University of Rome, 00185 Rome, Italy; 3 Department of Physiology and Pharmacology “Vittorio Erspamer”, Sapienza University of Rome, 00185 Rome, Italy; cristian.ieno@uniroma1.it (C.I.);; 4 Department of Occupational Medicine, Epidemiology and Hygiene, INAIL, Monte Porzio Catone, 00078 Rome, Italy; 5 R&D Aureli Mario S. S. Agricola, Via Mario Aureli 7, 67050 Ortucchio, Italy

**Keywords:** red beetroot juice, food intake biomarkers, metabolic profiling, NMR-based metabolomics, physical activity

## Abstract

Background: Red beetroot is known to be a health-promoting food. However, little attention is placed on intestinal bioactive compound absorption. The aim of the study was to assess the urinary red beetroot juice (RBJ) intake biomarkers and possible differences in RBJ’s micronutrient absorption at rest or after physical exercise. Methods: This is a three-armed, single-blind study, involving seven healthy volunteers which were randomly divided into three groups and alternatively assigned to three experimental sessions: RBJ intake at rest, RBJ intake with physical activity, and placebo intake with physical activity. For each session, urine samples were collected before and 120, 180, and 240 min after the intake of RBJ or placebo. The same sampling times were employed for the experimental session at rest. The RBJ metabolic composition was also characterized to identify the urinary biomarkers derived from the intake. Results: 4-methylpyridine-2-carboxylic acid, dopamine-3-O-sulfate, glutamine, and 3-hydroxyisobutyrate were identified as RBJ intake biomarkers. Physical activity significantly increased only the dopamine-3-O-sulfate excretion 120 min after RBJ intake. Conclusions: Urinary dopamine-3-O-sulfate is related to RBJ dopamine content, while 4-methylpyridine-2-carboxylic acid is a betanin or betalamic acid catabolite. The different excretions of these metabolites following physical activity suggest a possible effect on the RBJ uptake depending on different transport processes through the mucosa, namely diffusion-mediated transport for dopamine and saturable transcellular transport for betalamic acid derivatives. These results open new perspectives in improving the absorption of natural bioactive molecules through physical activity.

## 1. Introduction

Red beetroot (RB), *Beta vulgaris* L. subsp. *vulgaris* (*conditiva*), is characterized by high contents of nitrates, flavonoids, vitamins, minerals such as potassium, sodium, phosphorous, calcium, magnesium, copper, iron, zinc, and manganese, and the water-soluble pigments betalains, such as betacyanins (red-violet color) and betaxanthins (yellow orange color), all of which have numerous nutritional and health benefits [1]. RB is a significant source of phenols, which together with the betalains show a high antioxidant effect and radical scavenging capacity [2], an increase in the resistance of low-density lipoproteins (LDL) to oxidation, and the prevention of cancer and cardiovascular diseases [3]. Betalains constitute the major pigment of red beetroot and are composed of two major structural units, namely, red-violet betacyanins, including betanin, the main betacyanin present in red beetroot, and yellow-orange betaxanthins [4]. The structure of betalains is composed of a common unit of betalamic acid linked to cyclo-dopa or dopamine, derived from L-dopa metabolism.

Interestingly, a consistent amount of dopamine has been found in fresh red beetroot or in juice [5].

RB is also known to increase the blood levels of nitric oxide (NO), which is involved in multiple functions related to increased blood flow, gas exchange, mitochondrial efficiency, and strengthening of muscle contractions [6].

All these findings have suggested RB as an ideal and healthy supplement in the field of sports and physical activity for the improvement of physical fitness [6,7]. However, the health effect of micronutrients in red beetroot strongly depends both on their intake and on their bioavailability, which are key factors regarding functional foods and health claims related to food components [8]. In addition, nutrient absorption can vary depending on, among other things, physiological states and gut microbiota composition [9].

At the same time, it has been shown that exercise exerts positive effects on several aspects of health, such as sarcopenia [10] and inflammation reduction [11], also inducing positive changes in gut microbiota composition and in microbial metabolism [12,13]. Furthermore, moderate aerobic exercise is shown to promote muscle adaptations, lowering oxidative stress [14].

Consequently, for the latter, the absorption of different nutrients with exercise is expected even if the interaction between food and physical activity is very complex, also considering the scarce information on bioavailability of several bioactive compounds.

In this regard, a better understanding of the digestive fate of bioactive food compounds should allow us to unravel the health promotion and the performance effects of physical activity [8].

The aim of this study was to assess the effects of submaximal physical activity on the absorption of RB juice (RBJ) metabolites in healthy subjects. The urinary RBJ intake biomarkers, which are the metabolic products of characteristic molecules present in RBJ, were assessed, and metabolic profiles were characterized through NMR-based metabolomics at rest and after exercise.

## 2. Materials and Methods

### 2.1. Red Beetroot Juice Pretreatment for NMR Analysis

The subjects involved in this study drank 200 mL of commercial RBJs, which were provided by Aureli Mario S.S. Agricola and were processed in their industrial facility from beets collected in the Fucino fields of Abruzzo, Italy.

First, 1.5 mL of juice was extracted following a modified Bligh–Dyer protocol [15]. A cold mixture composed of chloroform, methanol, and water in a 2:2:1 proportion was added to each aliquot. After an overnight incubation at 4 °C, the samples were centrifuged for 25 min at 4 °C with a rotation speed of 10,000× *g*. The upper hydrophilic phase and the lower lipophilic phase were carefully separated and dried under a gentle flow of nitrogen. The hydrophilic phase was resuspended in D_2_O containing 3-(trimethylsilyl)-propionic-2,2,3,3-d4 acid sodium salt (TSP) 2 mM as an internal chemical shift and concentration standard. All solvents and standards were purchased from Sigma Aldrich (St. Louis, MO, USA).

### 2.2. Analysis of Red Beetroot Juice Samples by NMR Spectroscopy

All spectra were recorded at 298 K on a JEOL JNM-ECZR (JEOL Ltd., Tokyo, Japan) spectrometer equipped with a magnet that works at 14.09 T and at 600.17 MHz for the ^1^H frequency. ^1^H NMR spectra were acquired employing the presaturation pulse sequence for solvent suppression, with a spectral width of 9.03 KHz and 64 k data points for an acquisition time of 5.81 s. The employed pulse length was 8.3 μs corresponding to a flip angle of 90°, while the presaturation time length was 2 s. The recycle delay was set to 5.72 s to achieve complete resonance relaxation between subsequent scans. Bidimensional NMR experiments, ^1^H−^1^H TOCSY, ^1^H−^13^C HSQC, and ^1^H−^13^C HMBC, were performed on a few samples in order to allow univocal signal assignment and to identify the less superimposed resonances for each molecule to achieve the best selectivity and accuracy of quantification.

Quantities were expressed in mmol/mL through comparison of the relative integrals with the reference concentration and normalized to the number of protons (TSP: 9 protons) and to the starting milliliter of sample. The final concentration was expressed as mg/100 mL.

### 2.3. Characteristics of the Subjects and Experimental Setting

The group studied consisted of 7 healthy volunteers, 5 females and 2 males, non-smokers, aged 26.9 ± 3.0 years, mean BMI 22.0 ± 1.5 kg/m^2^. None of the subjects took dietary supplements or drugs potentially able to influence inflammatory status, nor other foods other than red beetroot juice during the time of the experiment. Subjects were asked to adhere to the Mediterranean dietary model and refrain from physical activity during the week before the experimentation. Only a light breakfast and drinking water were allowed during the intervention. Originally ten volunteers were included, however, three subjects dropped out.

The study was three-armed, randomized, and conducted in a single-blind crossover, with day A, B, and C indicating only the performed activity and not the consecutive days.

**Figure 1:**
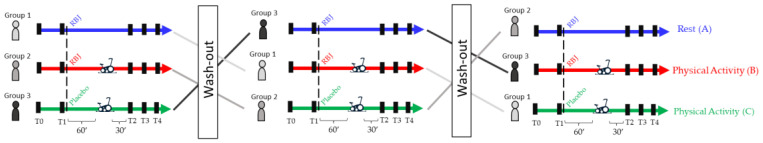
Urine sampling and intake of beetroot juice and placebo in the three experimental sessions.

The volunteers were randomly numerated from 1 to 10 and divided into three groups (group 1: numbers 1, 3; group 2: numbers 4, 7; and group 3: numbers 8, 9, 10) and alternatively assigned to the three experimental sessions with one week of wash-out, following the scheme reported in Figure 1.

In experimental session A, subjects took the RBJ at rest. In experimental session B, the subjects took the juice and performed their 30 min scheduled physical activity with a cycle ergometer, while in experimental session C they performed the same physical activity after the consumption of a placebo, which was composed of 7 g of sugar, 180 mL of water, and 20 mL of RBJ, making the placebo visually more similar to RBJ and hence not recognizable.

The subjects performed a scheduled exercise with a cycle ergometer both 1 h after the RBJ intake (session B) and 1 h after placebo intake (session C).

For each experimental session, five time points were considered for urine sample collection: T0, first urine in the morning at fast; T1, immediately before RBJ or placebo intake; T2, 120 min after the RBJ or placebo intake; T3, 180 min after RBJ or placebo intake; and T4, 240 min after the intake.

The study protocol conformed to the ethical guidelines of the 1975 Declaration of Helsinki, and it was approved by the Region Abruzzo Ethical Committee, ASL1 L’Aquila and Teramo districts, VERBALE—n° 23/CE/2016. All subjects provided their written informed consent to participate in the study.

### 2.4. Subjects’ Physical Profile and Exercise Workload Assessment

All subjects visited the laboratory to perform an incremental test on a bicycle ergometer (Ergoline 900, Cosmed, Albano Laziale (RM), Italy) in order to estimate both ventilatory thresholds (VT1 and VT2). During the test, gas exchange data were collected continuously using an automated breath-by-breath system (K5, Cosmed, Italy). The measuring instrument was calibrated before each test and the necessary environmental adjustments were made. The test protocol was composed of a rest phase 5 min long, where subjects remained seated on a bike without pedaling or other movements to assess basal parameters, a warm-up phase 3 min long, which consisted of pedaling with a power of 25 W, and an incremental phase, where pedaling was kept constant in a range of 70–80 rpm and the bike power increased by 5 W every 15 s. The incremental test was terminated (i) voluntarily by the subject, (ii) when pedaling cadence could not be kept firmly within range, or (iii) if in two successive load increments the VCO_2_/VO_2_ ratio exceeded 1.0. Only in this last case were the conditions of a correct ventilatory threshold estimation guaranteed. In all subjects the test was stopped with (iii).

VT1 was determined using the criteria of an increase in the ventilatory equivalent for oxygen (VE/VO_2_) with no increase in the ventilatory equivalent for carbon dioxide (VE/VCO_2_) and the departure from linearity of VE, whereas VT2 was determined by using the criteria of an increase in both VE/VO_2_ and VE/VCO_2_ [16]. Two independent observers detected VT1 and VT2 following the criteria previously described. If they did not agree, the opinion of a third investigator was included.

The purpose of the preliminary session was to determine the individual workload to also collect each subject’s measurements needed in the experimental sessions, including saddle height and pedal position. Participants started cycling at 30 W and, after 3 min, workload was increased to the individual’s pre-assessed VT1 (aerobic workload) and maintained for 30 min. This workload had to have the characteristics of being predominantly aerobic and being able to be sustained by the subjects throughout the session. Subjects maintained pedal rates of 70–80 rpm throughout all the sessions (B and C). Participants wore individual outfits and were allowed to drink during the experimental session. Temperature and humidity of the laboratory were kept constant during all sessions.

### 2.5. Urine Sample Preparation

For a proper metabolomic analysis of urine, sodium azide 0.05% *v/v* was added to the Falcon tubes (15 mL) employed for the urine collection, for a total sample volume of 10 mL. For each sample, 1200 μL of urine was collected and centrifugated at 11,000× *g* for 15 min at 4 °C to remove the cellular debris. Then, 1000 μL of supernatant was collected and added to 100 μL of an internal standard, sodium trimethylsilyl propionate in deuterated water (TPS-D_2_O, final concentration 2 mM). All the samples were adjusted to pH = 7 with a small addition of HCl or NaOH 0.1 N for resonance reproducibility and stored at −80 °C until the NMR analysis.

### 2.6. Analysis of Urine Samples by NMR Spectroscopy

NMR spectra were obtained at 298 K using a JEOL JNM-ECZR (JEOL Ltd., Tokyo, Japan) spectrometer equipped with a magnet that works at 14.09 T and at 600.17 MHz for the ^1^ H frequency. Each spectrum was registered with 64 k points and 64 scans, with a spectral width at 9.03 KHz (15 ppm), presaturation impulse duration of 2.00 s, and a relaxation delay of 5.72 s, for an acquisition time of 5.81 s. The identification phase was conducted through bidimensional experiments (^1^H-^1^H TOCSY and ^1^H-^13^C HSQC) applied to the selected sample, and with confirmation coming from bibliography comparison and databases. TOCSY experiments were recorded at 298 K with a spectral width of 15 ppm in both dimensions, using an 8 k × 256 data point matrix, repetition time of 3.00 s and 80 scans, and with a mixing time of 80.00 ms. HSQC experiments were acquired with a spectral width of 9.03 KHz (15 ppm) in the proton dimension and 30 KHz (200 ppm) in the carbon dimension, using an 8 k × 256 data point matrix for the proton and the carbon. The 2D-NMR spectra were processed by using JEOL Delta V5.3.1 software (JEOL Ltd., Tokyo, Japan).

^1^H-NMR spectra were analyzed using ACD ^1^H-Manager version 11.0 software (Advanced Chemistry Development, Inc., Toronto, ON, Canada), while bidimensional spectra were analyzed with JEOL Delta software. An exponential window function with a line-broadening factor of 0.30 Hz was applied to the FIDs before being Fourier transformed. Each of the spectra thus obtained were then manually phased and baseline corrected. The reference signal from the TSP methyl resonance was set to 0.00 ppm.

The quantification of the metabolites was obtained by comparing the integrals of their diagnostic resonances with the internal standard TSP integral and normalized for their number of protons. Data from urine spectra were normalized to the methyl peak integral of creatinine at 4.05 ppm to correct for dilution effects and expressed as μmol/mmol of creatinine (Supplementary Table S3).

### 2.7. Data Analysis and Statistics

Multivariate and univariate statistical analyses were applied in order to evaluate the metabolic differences related to physical activity and RBJ intake.

Principal component analysis (PCA) was applied for exploratory purposes to identify clusters and outliers. The data matrix was previously auto-scaled and centered before further processing. For the classification stage, models were built using the partial least square-discriminant analysis (PLS-DA) algorithm. The full-cross validation method was applied, and the area under receiver operating characteristic (AUROC), R^2^, and Q^2^ were employed as diagnostic statistics for assessing the predictive ability and the quality of the classification models.

The multivariate analysis was carried out by using Unscrambler 10.5 software (CAMO, Oslo, Norway). AUROC was calculated with an in-house written script employing MATLAB R2020b.

Univariate evaluation of the variables identified as significant based on PLS-DA analysis was applied by using the paired Student’s *t*-test or Wilcoxon signed rank sum test for paired data according to the Shapiro–Wilk normality test and two-way ANOVA for multiple comparison using Bonferroni correction. A *p*-value of 0.05 was considered as the threshold for statistical significance. Sigmaplot 14.0 Systat Software Inc. (Palo Alto, CA, USA) was employed for univariate statistical analysis. 

## 3. Results

### 3.1. Red Beetroot Juice Composition

The chemical composition of the commercial RBJ was assessed by NMR spectroscopy in order to identify the metabolites which could act as substrates or could be absorbed in the intestinal mucosa in relation to the intake. For this purpose, five replicates of hydroalcoholic extracts of RBJ were analyzed, and a representative spectrum of red beetroot hydroalcoholic extract is reported in Supplementary Figure S1. A total of 29 metabolites were quantified from the ^1^H NMR spectra of the hydroalcoholic extract. The ^1^H chemical shifts and multiplicity of the identified molecules are reported in Supplementary Table S1. The metabolite concentrations, expressed as mean and standard deviation, are reported in Table 1.

The analysis of the hydroalcoholic extracts of beetroot juice showed a considerable amount of carbohydrates (total amount: 6174 ± 454 mg/100 mL), amino acids (total amount: 427 ± 7 mg/100 mL), and some bioactive molecules, including betaine, betanin, dopamine, and other antioxidant molecules.

### 3.2. Experimental Setting

The urine samples of seven volunteers were collected to determinate the urinary metabolomic profile correlated to physical activity and the consumption of 200 mL of red beetroot juice (RBJ). The experiment was carried out in three different experimental sessions (A, B, and C), with a common controlled food intake at breakfast.

The exercise consisted of 3 min of warm-up at 30 W, followed by 30 min of aerobic exercise at constant workload at the individual pre-assessed VT1 (see Materials and Methods section). The subjects maintained pedal rates between 70–80 rpm. The volunteers were randomly numerated from 1 to 10 and divided into three groups (group 1: numbers 1, 3; group 2: numbers 4, 7; group 3: numbers 8, 9, 10) and alternatively assigned to the three experimental sessions with one week of wash-out. For each subject, five samples of urine were collected on each day at different times: first urine in the morning at fast (T0), one hour before exercise or rest (T1), and 120 min (T2), 180 min (T3), and 240 min (T4) after drinking the juice or placebo (Figure 1). All the subjects took the RBJ or placebo immediately after T1. For the detailed experimental setting, see the Materials and Methods section.

### 3.3. Urinary Metabolomics

A representative urinary ^1^H-NMR spectrum and the detailed regions are reported in Supplementary Figures S2–S4. The resonance assignment is reported in Supplementary Table S2, and a total of thirty-six metabolites were identified and quantified from ^1^H-NMR spectra.

### 3.4. Urinary Biomarkers of RBJ Intake

In order to identify the urinary biomarkers of RBJ intake, we identified and quantified molecules whose resonances were observed in ^1^H-NMR spectra collected in experimental session A (at rest and after the RBJ intake), considering sampling times from T1 to T3 (Figure 1). Two metabolites resulted as crucial in the discrimination between the urine samples and were identified as urinary biomarkers of RBJ intake: 4-methylpyridine-2-carboxylic acid (4MP2CA) and dopamine-3-O-sulfate (DA3S). Their resonances and structures are reported in Table 2.

**Figure 2:**
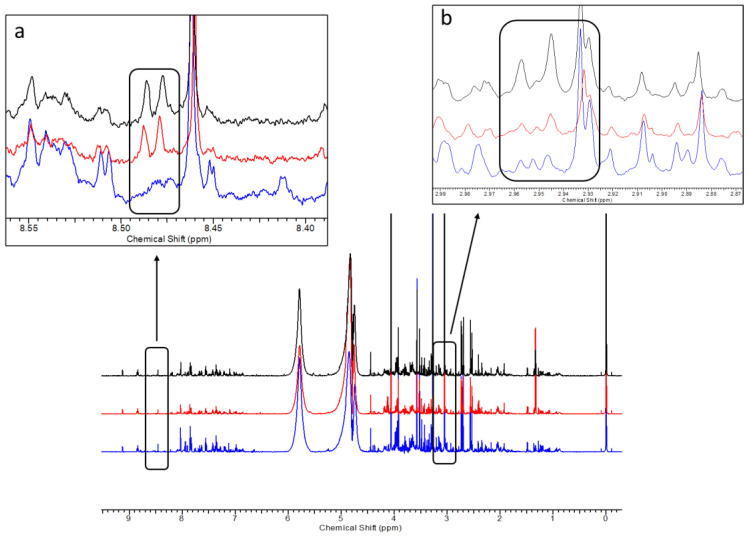
From the bottom to the top, ^1^H NMR urinary spectra of one subject collected at T1 (blue), T2 (red), and T3 (black) in session B for diagnostic signals of (**a**) 4-methylpyridine-2-carboxylic acid and (**b**) dopamine-3-O-sulfate.

Comparing the urinary spectra of subjects at rest, it was indeed possible to observe both quantitative and qualitative differences in these molecules observable only after the RBJ intake, as reported in Figure 2a,b for day A, before the juice intake, and at two (T2) and three hours (T3) after the juice intake. Furthermore, those signals were not detectable in spectra of urines from the placebo group (Supplementary Figure S5).

**Figure 3:**
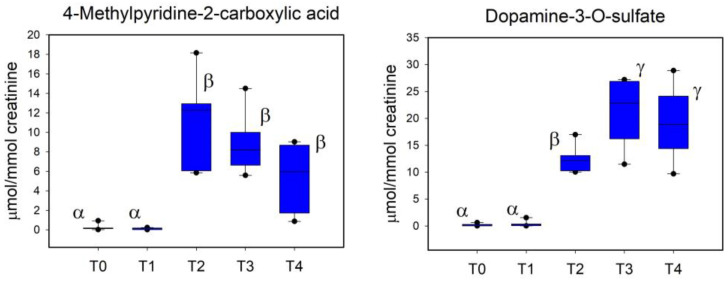
Boxplot of 4-methylpyridine-2-carboxylic acid and dopamine-3-O-sulfate for all the individuals across the sampling time for experimental session A. Statistical significance was assessed by two-way ANOVA—all pairwise multiple comparison procedures (Bonferroni *t*-test). A *p*-value of 0.05 was considered as the threshold for statistical significance, which is reported as Greek letters (α or β or γ) in the boxplots. The same letters indicate that there is no statistically significant difference; different letters indicate that there is a statistically significant difference among groups.

**Figure 4:**
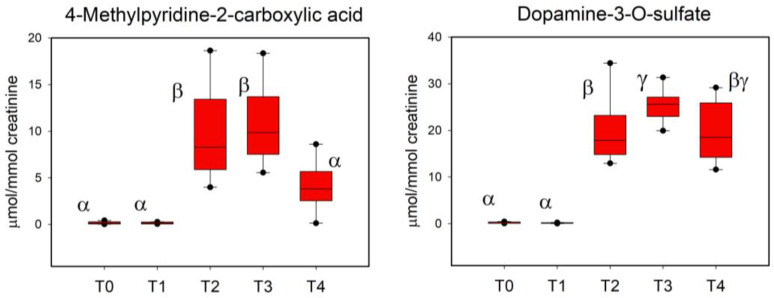
Boxplot of 4-methylpyridine-2-carboxylic acid and dopamine-3-O-sulfate for all the individuals across the sampling time for experimental session B. Statistical significance was assessed by two-way ANOVA—all pairwise multiple comparison procedures (Bonferroni *t*-test). A *p*-value of 0.05 was considered as the threshold for statistical significance, which is reported as Greek letters (α or β or γ) in the boxplots. The same letters indicate that there is no statistically significant difference; different letters indicate that there is a statistically significant difference among groups.

In order to investigate if the physical activity could have an effect on the urinary excretion of the identified RBJ biomarkers, a univariate statistical analysis on the quantitative data by comparing metabolite levels from T0 to T4 at rest and with exercise, with the RBJ intake being equal (sessions A and B), was carried out. The same RBJ biomarkers of Figure 2 but with level variations over time are reported in Figure 3, session A, and Figure 4, session B. For session A, an increase in 4MP2CA excretion was detected at T2, with a subsequent slow decrease at T3 and T4 (Figure 3). On the contrary, a sharp increase in DA3S levels was observed at T3, with a subsequent decrease at T4, while maintaining a statistical significance with respect to both T0 and T1, and to T2 (Figure 3).

For session B, an increase in 4MP2CA excretion was detected at T2, with a subsequent increase at T3, though not statistically significant, and a significant decrease at T4, tending to return at T0–T1 levels (Figure 4). At the same time, a strong increase in DA3S levels was observed at T2, reaching a maximum at T3, with a subsequent slow decrease at T4, not statistically significant with respect to T2 and T3 (Figure 4).

In order to investigate if statistically significant differences in RBJ biomarker urinary excretions could be observed related to physical activity, we applied a paired Student’s t-test on each sampling time for sessions A and B.

**Figure 5:**
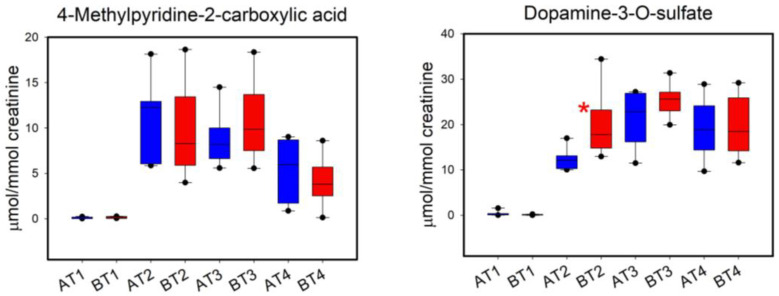
Boxplot of 4-methylpyridine-2-carboxylic acid and dopamine-3-O-sulfate at T1, T2, T3, and T4 for days A (blue) and B (red). Red asterisk indicates a statistically significant difference, according to paired Student’s *t*-test (*p* < 0.05) performed between subjects at session A and session B.

As reported in Figure 5, there is not a significant variation in 4MP2CA between A and B for all the considered times. On the contrary, there is a statistically significant increment of DA3S between A and B for T2. A similar trend is also observed for T3, even though it did not reach statistical significance.

In order to evaluate urinary metabolic changes linked to the RBJ intake and physical activity, a PCA analysis including all the subjects was performed (Supplementary Figure S6) on the entire dataset. The score plot did not show a clear grouping according to both aforementioned factors, even though a slight separation with respect to the individuals was observed. Since the PCA model evidenced a high inter-individual variability, we proceeded to apply a supervised model (PLS-DA) in order to maximize the differences due to a specific factor (RBJ intake and physical activity).

### 3.5. Metabolic Profile Related to Physical Activity

**Figure 6:**
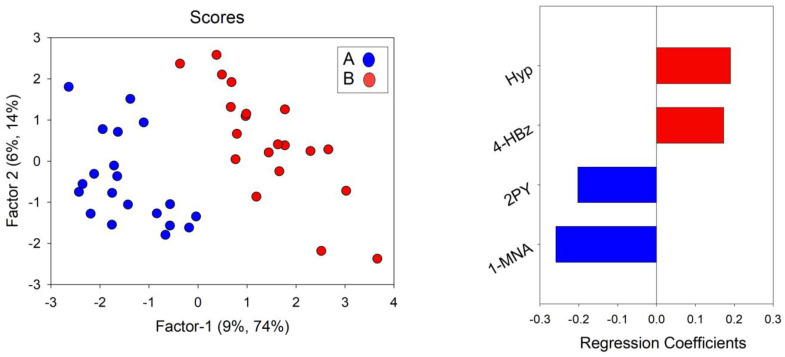
PLS-DA performed between subjects at day A (blue) and day B (red): **left-side**—score plot; **right-side**—regression coefficient values reported as horizontal histograms. In blue are reported the metabolites which are higher on day A, while in red are reported the metabolites which are higher on day B.

In order to assess the effect of physical activity on the urinary profile, we applied a PLS-DA model on the data matrix composed of samples from T2 to T4 collected in sessions A (at rest) and B (physical activity) (Figure 6). The analysis provided a robust two-component model (R^2^ = 0.88 and Q^2^ = 0.74), with AUROC = 0.75 for the specificity and sensitivity of the model (Supplementary Figure S7). On the basis of the regression coefficient values (Figure 6), it is possible to observe that the physical activity increased the levels of 4-hydroxybenzoate (4-HBz) and hypoxanthine (Hyp) and lowered the those of N1-methyl-2-pyridone-5-carboxamide (2PY) and 1-methylnicotinamide (1-MNA).

**Figure 7:**
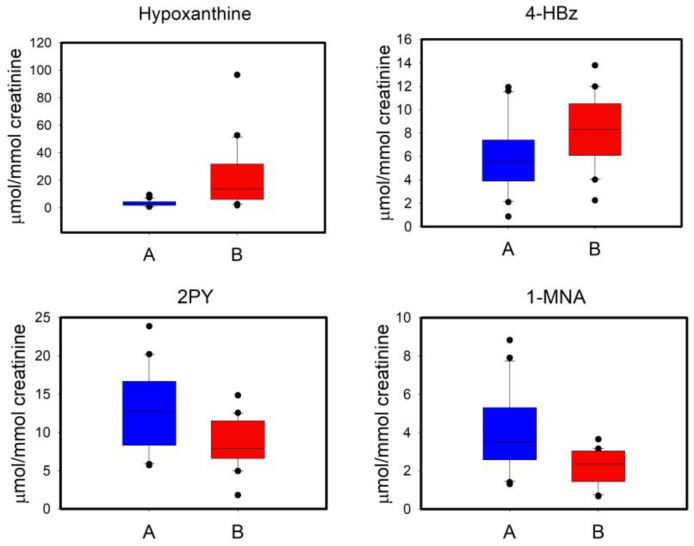
Boxplot of urinary biomarkers related to physical activity. A (blue) indicates experimental session at rest, B (red) indicating experimental session with physical activity, being equal the RBJ intake.

The observed differences in 4-hydroxybenzoate (4-HBz), hypoxanthine, 1-methyl-2-pyridone-5-carboxamide (2PY), and 1-methylnicotinamide (1-MNA) levels resulted to be also significant according to the Wilcoxon signed rank test (Figure 7).

### 3.6. Metabolic Profile Related to Red Beetroot Juice Intake

**Figure 8:**
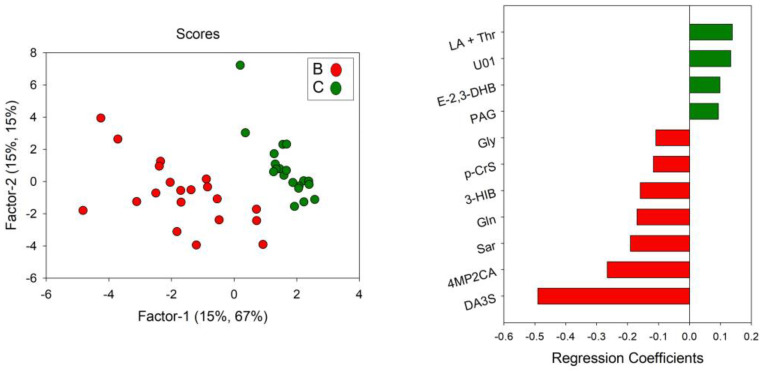
PLS-DA performed between subjects on day B (red) and day C (green): **left**-side—score plot; **right**-side—regression coefficient values reported as horizontal histograms. In red are reported the metabolites which are higher on day B, while in green are reported the metabolites which are higher on day C.

In order to evaluate if RBJ intake could have an effect on the urinary metabolic profile, a PLS-DA was performed on the data matrix comparing subjects on day B (physical activity with RBJ) to the same subjects on day C (physical activity with placebo). Sampling times from T2 to T4 were considered all together. The metabolic profiles of the two classes were well separated by inspecting the PLS-DA score plot (Figure 8). The PLS-DA model resulted in five significant latent variables (LVs), R^2^ = 0.95, and Q^2^ = 0.77. The AUROC was 0.72 (Supplementary Figure S8). On the basis of regression coefficient values, 11 metabolites were identified as significantly relevant for the discrimination. In particular, the levels of 3-hydroxyisobutyrate (3-HIB), p-cresol sulfate (p-CrS), glutamine (Gln), sarcosine (Sar), DA3S, glycine (Gly), and 4MP2CA were higher on day B, while the levels of lactate + threonine (LA + Thr), erythro-2,3-dihydroxybutyrate (E-2,3-DHB), phenylacetylglycine (PAG), and an undefined metabolite (U01) were higher in subjects who took the placebo (day C) than in those who took the RBJ (day B).

**Figure 9:**
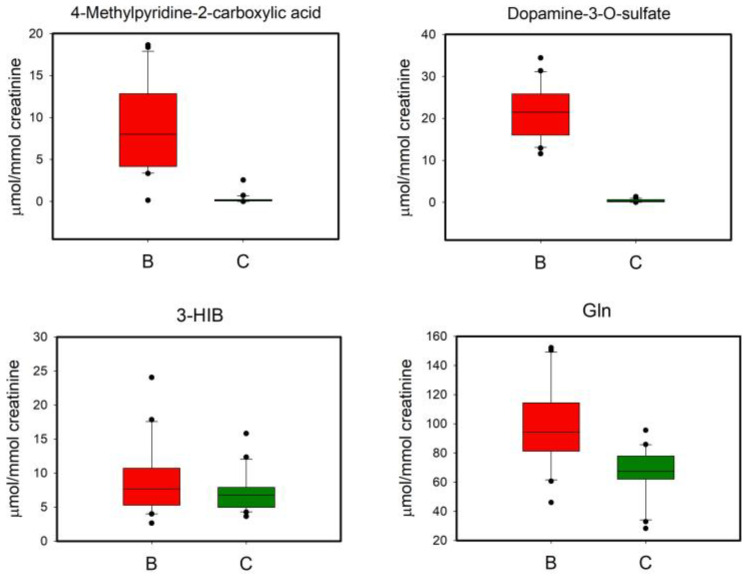
Boxplot of urinary biomarkers related to red beetroot juice intake. B (red) indicates RBJ intake, C (green) indicates placebo intake, being equal the physical activity.

The differences in levels of Gln, 3-HIB, DA3S, and 4MP2CA observed between subjects on day B and day C resulted to also be significant (*p* < 0.05) by the Wilcoxon signed rank test, as reported in Figure 9.

## 4. Discussion

The identification of new biomarkers of food and nutrient bioavailability has developed fast over the past two decades and could potentially provide new tools for dietary intake assessment. In this context, metabolomics has opened new opportunities, since new putative biomarkers are frequently identified by the metabolic profiling of body fluids following the ingestion of several foods [17].

To the best of our knowledge, this study is the first NMR-based study that identified and quantified the urinary biomarkers of red beetroot juice intake and comprehensively investigated the combined effect of red beetroot juice and physical activity on healthy individuals.

This is a pilot study, and the number of subjects was sufficient for a crossover experimental design. Moreover, we examined the same group in several different conditions and time points, for a total of fifteen urine samples for each volunteer.

In agreement with a previous mass-spectrometry study, DA3S and 4MP2CA were the identified urinary biomarkers related to RBJ intake [18]. Our results showed that these molecules derive from bioactive compounds present in RBJ, namely dopamine and betalamic acid, respectively. The range of dopamine levels was from 7 to 10 mg in the red beetroot juice, and it is a strong hydrosoluble antioxidant. However, the uptake of dopamine at the intestinal level is very hindered by the gut mucosa barrier [19].

Sulfate conjugation is an important pathway for the detoxication of xenobiotics, and previous studies have shown that in plasma more than 98% of dopamine is sulfated [20]. This process has been proposed to play important roles in the regulation and biotransformation of catecholamines [21].

The sulfonation of phenolic hydroxyl groups is catalyzed by the SULT1 family, which is a group of cytosolic sulfotransferases present in the intestine epithelium, liver, and kidneys [22,23]. Furthermore, SULT1A3 is shown to sulfate catecholamine neurotransmitters and is abundant in ileal and colonic mucosa [24]. Moreover, it has been also reported that the microbiota-mediated sulfation process could also occur in the gut. However, an independent study performed by our laboratory demonstrated the absence of DA3S in the stool of 32 subjects after taking RBJ at rest. The presence of dopamine sulfate in urine is in agreement with a diffusion-mediated transport of dopamine from the intestinal lumen to the cytosol of epithelial cells and with the intracellular sulfation of dopamine. Then, DA3S can move into the bloodstream and be excreted in urine [19].

The present results showed a significant increment of urinary DA3S two times higher at T2 comparing exercise and rest, with the RBJ intake being equal. Furthermore, the level of DA3S increased, peaking at T3.

DA3S is an endogenous metabolite, but a 20-fold increment was observed comparing the RBJ intake with placebo intake, with the physical activity intensity being equal. These results were in agreement with the increase in dopamine sulfate observed in cyclists exercising after banana intake, contributing to observed increment of antioxidant capacity [25]. It is noteworthy that banana pulp has about 3–10 mg of dopamine per 100 g of pulp, like red beetroot [26]. Furthermore, the exercise increased the rate of appearance of the urinary dopamine sulfate through a mechanism not yet understood, probably related to an undirected effect due to the peculiar transport properties of the specific molecules.

Interestingly, at T3 the urine of all subjects was colored, indicating the presence of a coloring molecule, probably betanin. This datum resulted more intense after physical activity than at rest and was not recognized during the placebo experiment (Supplementary Figure S9). Unfortunately, the levels of betanin were not measurable by NMR spectroscopy, but the increase in red color in urine was observed at the same time of the peak of dopamine sulfate. It has been shown that 65% of betanin crossed the intestinal epithelial cells’ monolayer by paracellular passive diffusion, indicating that it can reach the bloodstream in its active form [3]. The urinary betanin content has been reported to be between 0.3–0.9% of the ingested dose via 300 mL of RBJ [27,28], a concentration well below the sensitivity limit of NMR spectroscopy.

**Figure 10:**
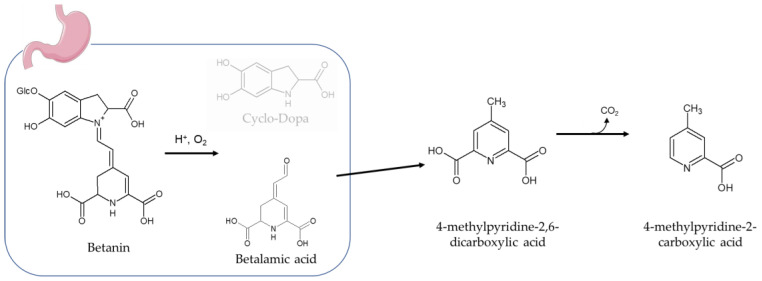
Possible metabolic fate of betanin in the stomach.

4MP2CA, the other suggested biomarker of RBJ intake, is a decarboxylation product of 4-methylpyridine-2,6-dicarboxylic acid, a catabolite of betalamic acid produced in the gastrointestinal tract (Figure 10) [28]. More than 50% of oral administered betanin is decomposed by stomach gastric acid in betalamic acid and cyclo-dopa [29].

The degradation of betanin by the decarboxylation route was found to be dependent on heating, pO_2_, and pH [30]. Our NMR spectroscopy study on the RBJ did not find specific signals of decarboxylated betanin compounds or 4MP2CA, hence excluding their production in the RBJ transformation process. Furthermore, the NMR analysis highlighted the low concentration of free betalamic acid in the juices. Therefore, the levels of 4MP2CA could only be derived from the decomposition of betanin or of betalamic acid at the gastrointestinal level.

**Figure 11:**
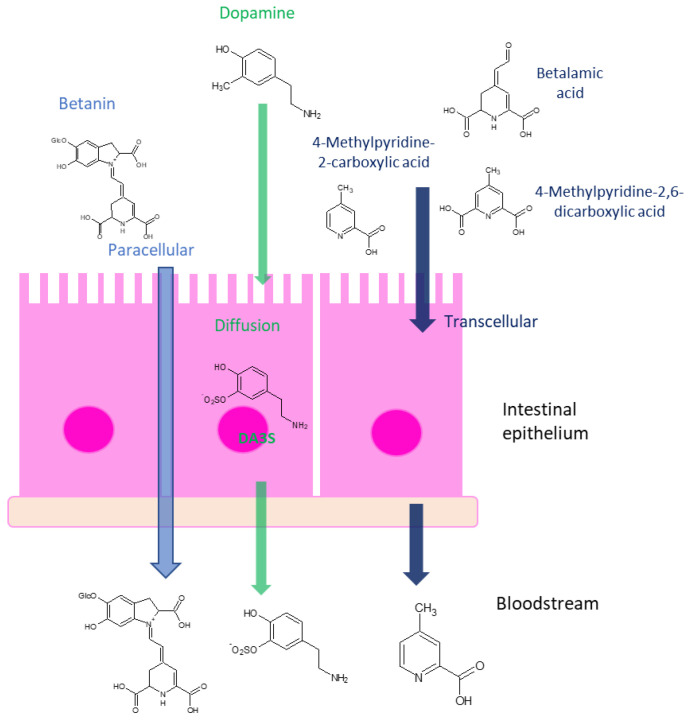
Physiologic model of intestinal absorption for dopamine, betanin, and 4-methylpyridine-2-carboxylic acid.

The transport through the intestinal mucosa of 4-methylpyridine-2,6-dicarboxylic acid or 4MP2CA is not known, but a transcellular transport could be hypothesized, analogous to that reported for pyridine compounds mediated by organic anion transporters or monocarboxylate transporters [31,32]. Our experiments did not reveal any significant variations in 4MP2CA acid at T2, T3, and T4 after exercise compared with rest. The different transport mechanisms suggested for betalamic acid derivatives (saturable transcellular transport) and dopamine and betanin (paracellular passive diffusion) could explain the lower effect of the exercise on the urinary excretion of 4MP2CA with respect to DA3S. During aerobic exercise, an increase in urinary markers that indicate augmented intestinal permeability can occur [33]. In fact, an increase in sympathetic system stimuli can lead to alterations in intestinal motility and absorption capacity [34,35,36]. This could result in the increased excretion of either DA3S (colorless) or betanin (red color), as observed in urine coloration (Supplementary Figure S9). Therefore, the effect of exercise on the levels of DA3S and betanin could be explained on the basis of a higher bloodstream extraction from intestinal mucosa in the presence of a high gradient of dopamine and betanin concentration between the lumen and basal membrane, also in the presence of a transient modified paracellular transport (Figure 11).

The urinary metabolic profile of RBJ with respect to placebo intake was also characterized by higher levels of 3-HIB and Gln, with the workload exercise being equal.

The biomarker 3-HIB is a biomarker for valine catabolism in skeletal muscle [37], and its elevation after physical activity is largely reported in the previous literature [38,39]. Higher 3-HIB urinary excretion is in agreement with the valine supplementation in the RBJ (total amount of valine is 30.8 mg per 200 mL of juice, Table 1).

Regarding Gln, physical exercise can differentially affect Gln production in muscle and its availability in plasma, decreasing Gln availability following strenuous exercise [40]. Indeed, it has been shown that endurance and resistance sports characterized by prolonged and intense energy expenditure were associated with the worst decreases in glutamine availability [41]. Studies reported higher glutamine levels in carbohydrate-supplemented rats when compared with placebo [42], whereas in athletes fed low-carbohydrate diets, glutamine concentration was shown to be reduced [43]. RBJ contains a substantial amount of carbohydrates, both non-essential and essential amino acids, including a large amount of glutamine (Table 1). For this reason, Gln can be also considered a biomarker of juice intake, even if it is not a specific metabolite.

The metabolic profiles of volunteers after physical activity and RBJ intake showed significant variations in four metabolites compared to RBJ intake at rest. The variations were represented by an increase in Hyp and 4-HBz levels and a decrease in 1-MNA and 2PY levels.

Hyp is a nucleotide degradation product involved in purine metabolism. Purine metabolites are more sensitive markers of training status and better performance predictors than typical biochemical and physiological indicators [44]. It has been demonstrated that plasma Hyp concentration depends on exercise intensity. Hyp may be an index of muscle adenine nucleotide degradation and a marker of exercise-induced energetic stress. It has already been demonstrated that, in the context of particular physiological conditions such as physical activity, several reactive oxygen species (ROS) may be involved, since muscular activity has been shown to associate with ROS production [45].

In our study, the levels of Hyp significantly changed depending on exercise, but not on RBJ intake. In order to evaluate the possible effect of RBJ intake on muscular metabolism, further studies should be performed using a larger amount of juice intake or a higher intensity workload, as reported in other experimental protocols [46].

The metabolite 4-HBz is found to be a product of microbial phenol metabolism [47,48] and could account for substantial proportions of the ingested polyphenol doses [49]. Phenols are important compounds because of their contribution to human health and their multiple biological effects, such as antioxidant activity, antimutagenic and/or anticarcinogenic activities, and anti-inflammatory action [50].

After RBJ intake, it was found that 4-HBz was significantly higher after exercise compared to rest. After exercise, this metabolite remained unchanged both in RBJ and placebo intake. For all the above, it is possible to hypothesize that physical activity plays a role in the metabolism of aromatic compounds.

Urinary levels of 1-MNA and 2PY were significantly lower in subjects after physical activity compared to the same subject at rest, with the RBJ intake being equal.

At rest and at fast, a borderline negative correlation between 1-MNA and nicotinamide riboside (precursor of NAD+) was found (*n* = 21; ρ = −0.394; *p* = 0.076), whereas a significant positive correlation between 1-MNA and 2PY was observed (*n* = 21; ρ = 0.74; *p* < 0.0001). These findings are in agreement with the three different metabolic routes of nicotinamide: NAD+ salvage pathways, methylation, and nicotinamide degradation. The NAD+ salvage pathway generates NAD+ by converting nicotinamide and alpha-d-5-phosphoribosyl-1-pyrophosphate to nicotinamide mononucleotide (NMN) and inorganic pyrophosphate in a reaction catalyzed by nicotinamide phosphoribosyltransferase [51] (Kegg reference pathway hsa00760). The methylation produces 1-MNA from nicotinamide (NAM, vit B3) through nicotinamide N-methyltransferase (NNMT); finally, 2PY and 1-methyl-4-pyridone-5-carboxamide (4PY) are degradation products derived from the activity of aldehyde oxidases (AOX) [52,53]. NNMT is a cytosolic enzyme, mainly expressed in the liver and in adipose tissue, which catalyzes the N-methylation of NAM. NNMT regulates glucose, lipid, and cholesterol metabolism by stabilizing the sirtuins-1 (SIRT1) enzyme [54]. Physical exercise significantly changes metabolism, affecting the NAD^+^:NADH ratio and thus leading to a strong induction of SIRT1 activity [55]. Lower urinary concentrations of 1-MNA and 2PY after aerobic exercise in healthy volunteers could be due to a higher turnover of nicotinamide and nicotinamide riboside in the synthesis of NAD, with less urinary excretion of the final catabolites.

## 5. Conclusions

To the best of our knowledge, our study is the first NMR-based investigation that identified the RBJ intake biomarkers and evaluated their changes induced by physical activity. This study was not aimed at evaluating the metabolic effects of RBJ on performance, which has been often related to the high nitrate content of RBJ and to the physiological effect of nitric oxide. Our study showed a different behavior in the urinary excretion of the identified RBJ intake biomarkers in relation to physical activity. These differences could reflect the physiology of the gastrointestinal system, involving the splanchnic blood flux and/or intestinal permeability, and thus leading to a better passage of molecules. The possibility of influencing through physical activity the adsorption of plant bioactive compounds is very intriguing, since the intestinal transport of these molecules mainly occurs via diffusion processes or paracellular mechanisms.

This investigation opens a new perspective on the importance of food bioavailability for human health and athletic performance. It is evident that the effect could depend on the physical exercise intensity and should be further confirmed.

## Figures and Tables

**Table 1:** Mean and standard deviation (SD) of red beetroot juice metabolites expressed as mg/100 mL of juice, measured by nuclear magnetic resonance spectroscopy on the final product.

Class	Metabolite	Amount (mg/100 mL)
Amino acids	Alanine	90.7 ± 7.0
	Asparagine	29.0 ± 2.3
	GABA	11.95 ± 0.88
	Glutamate	155.9 ± 6.8
	Glutamine	80.2 ± 8.4
	Isoleucine	15.9 ± 1.2
	Leucine	7.81 ± 0.59
	Phenylalanine	0.572 ± 0.051
	Threonine	10.78 ± 0.86
	Tryptophan	2.65 ± 0.19
	Tyrosine	6.09 ± 0.47
	Valine	15.4 ± 1.2
Organic acids	Betalamic acid	0.763 ± 0.071
	Citrate	169 ± 14
	Formate	1.421 ± 0.092
	Fumarate	0.694 ± 0.051
	Malate	84.6 ± 7.3
	4-Hydroxybenzoate	0.393 ± 0.032
	4-Hydroxycumarate	1.054 ± 0.054
Carbohydrates	Arabinose	9.56 ± 0.78
	Fructose	161 ± 11
	Glucose	393 ± 31
	Sucrose	5610 ± 454
Miscellaneous	Betaine	245 ± 20
	Betanin	12.6 ± 1.1
	Choline	1.91 ± 0.18
	Dopamine	7.26 ± 0.65
	Myo-Inositol	147 ± 12
	Trigonelline	1.13 ± 0.11

**Table 2:** Resonance assignment of 4MP2CA and DA3S: s—singlet; d—doublet; t—triplet; dd—doublet of doublets. Bold indicated the resonances that have been taken into account for quantitation.

Molecular Portion	Structure	δ ^1^H	Multiplicity
4-Methylpyridine-2-carboxylic acid			
CH-3	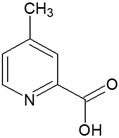	7.90	d
CH-5		7.48	dd
**CH-6**		**8.48**	**d**
CH_3_		2.46	s
Dopamine-3-O-sulfate			
CH_2_-α	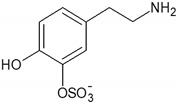	3.27	t
**CH_2_-β**		**2.94**	**t**
CH-2		6.92	d
CH-5		6.91	d
CH-6		6.75	dd

## Data Availability

Data are available as Supplementary Table S3.

## Supplementary Materials

The following supporting information can be downloaded at: https://www.mdpi.com/article/10.3390/nu15092026/s1, Figure S1: ^1^H NMR spectrum of red beetroot juice hydroalcoholic extract; Table S1: Assignment of red beetroot juice (RBJ) extract; Figure S2: ^1^H NMR urinary spectrum of a control sample; Figure S3: ^1^H NMR spectrum of urine, from 1 ppm to 4 ppm; Figure S4: ^1^H NMR spectrum of urine, from 6.5 ppm to 9 ppm; Table S2: Resonance assignment of urine; Figure S5: ^1^H NMR urine of one subject collected at day B (red) and day C (green) three hours after the juice intake (T3): (a) 4-methylpyridine-2-carboxylic acid; (b) dopamine-3-O-sulphate; Figure S6: Score plot of principal component analysis (PCA) performed on all the subjects, considering all five sampling times and all the experimental sessions; Figure S7: Area under receiver operating characteristics for PLS-DA between rest and physical activity, with the RBJ intake being equal (A vs. B); Figure S8: Area under receiver operating characteristics for PLS-DA between RBJ and placebo intake, with the physical activity being equal (B vs. C); Figure S9: Picture of urine collected from the same subject during A) the experimental session with placebo and B) the experimental session with RBJ intake, with the physical activity being equal; Table S3: Concentrations of urinary metabolites expressed as µmol/mmol of creatinine.
