# Supplementary material for: Metabolic Biomarkers of Red Beetroot Juice Intake at Rest and after Physical Exercise
Source: Nutrients. 2023 Apr 22;15(9):2026. doi: 10.3390/nu15092026 (PMC10180642; doi:10.3390/nu15092026)
Supplement: Supplementary file 1 [file nutrients-15-02026-s001.zip › Supplementary.pdf]

Metabolic intake biomarkers of red beetroot juice at rest and after physical exercise

Ottavia Giampaoli[1,2,†], Cristian Ieno[3,†], Fabio Sciubba[1,2], Mariangela Spagnoli[1,4], Alfredo Miccheli[1,2,*], Alberta Tomassini[5], Walter Aureli[5], Luigi Fattorini[3]

[1] NMR-based Metabolomics Laboratory (NMLab), Sapienza University of Rome, 00185 Rome, Italy; ottavia.giampaoli@uniroma1.it; fabio.sciubba@uniroma1.it; mariangela.spagnoli@uniroma1.it; alfredo.miccheli@uniroma1.it
[2] Department of Environmental Biology, Sapienza University of Rome, 00185 Rome, Italy;
[3] Department of Physiology and Pharmacology "Vittorio Erspamer", Sapienza University of Rome, 00185 Rome, Italy; cristian.ieno@uniroma1.it; luigi.fattorini@uniroma1.it;
[4] Department of Occupational Medicine, Epidemiology and Hygiene, INAIL, Monte Porzio Catone, 00078 Rome, Italy; m.spagnoli@uniroma1.it;
[5] R&D Aureli Mario S. S. Agricola, Via Mario Aureli 7, Ortucchio (Aq), 67050, Italy
* Correspondence: alfredo.miccheli@uniroma1.it
† These authors contributed equally to this work

Supplementary Material

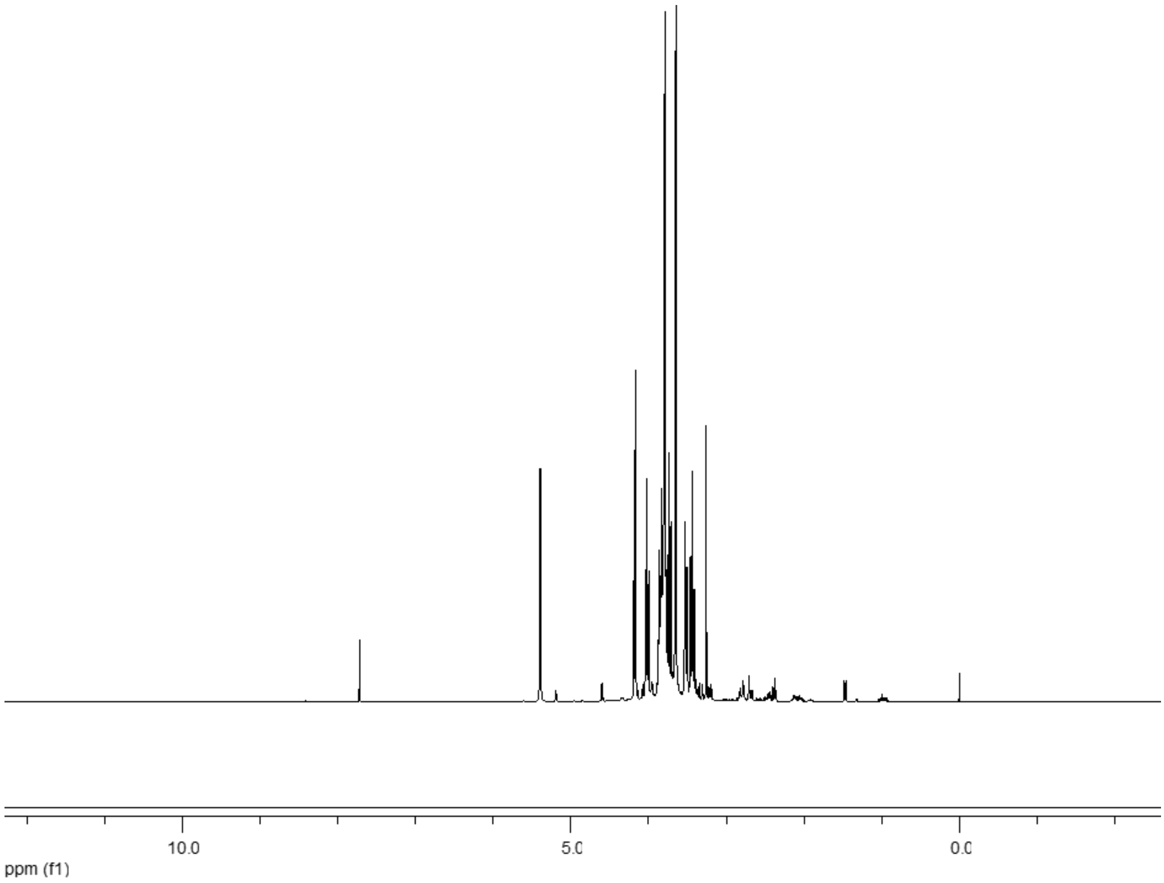

Figure S1. ^{1}H NMR spectrum of red beetroot juice hydroalcoholic extract.

Table S1. Assignment of red beetroot juice (RBJ) extract

Metabolite	^{1}H δ (ppm)	Multiplicity	Assignment
1. Leucine (Leu)	**0.97**	**m**	**δ,δ′-CH_3**
	1.72	m	γ-CH
	1.73	m	β-CH_2
	3.74	m	α-CH
2. Isoleucine (Ile)	0.95	t	δ-CH_3
	**1.02**	**d**	**γ-CH_3**
	1.25	m	γ′-CH
	1.49	m	γ′′-CH
	1.99	m	β-CH
	3.69	m	α-CH
3. Valine (Val)	0.99	d	γ-CH_3
	**1.05**	**d**	**γ′-CH_3**
	2.29	m	β-CH
	3.62	m	α-CH
4. Threonine (Thr)	**1.32**	**d**	**γ-CH_3**
	3.60	m	α-CH
	4.27	m	β-CH
5. Alanine (Ala)	**1.49**	**d**	**β-CH_3**
	3.80	q	α-CH
6. Acetic acid (AA)	**1.92**	**s**	**CH_3**
7. Gamma amino butyric acid (GABA)	1.91	m	β-CH_2
	2.35	t	α-CH_2
	**3.03**	**t**	**γ-CH_2**
8. Glutamate	2.09	m	γ-CH_2
	**2.34**	**m**	**β-CH_2**
	3.74	m	α-CH
9. Glutamine	2.11	m	γ-CH_2
	**2.45**	m	**β-CH_2**
	3.81	m	α-CH
10. Citric acid (CA)	**2.67**	**d**	**α,γ-CH**
	2.71	d	α',γ'-CH
11. Asparagine (Asn)	2.86	dd	β′-CH
	**2.95**	**dd**	**β-CH**
	4.01	m	α-CH
12. Choline (Chn)	**3.21**	**s**	**N-$(CH_3)_3$**
	3.51	t	CH_2
	4.07	t	CH_2
13. Betaine	**3.26**	**s**	**N-$(CH_3)_3$**
	3.84	s	CH_2
14. Fructose	3.69	m	CH-1
	\	\	C-2
	**4.22**	**d**	**CH-3**
	4.06	m	CH-4
	3.90	m	CH-5

	3.82	m	CH-6
15. Malate	**4.31**	**dd**	**α-CH**
	2.38,2.69	dd	β,β′-CH
16. β-Arabinose	**4.58**	**d**	**CH-1**
	3.92	m	CH-4
	3.65	m	CH-3
	3.50	m	CH-2
	3.67-3.88	m	CH_2-6
17. β-Glucose	**4.65**	**d**	**CH-1**
	3.26	m	CH-2
	3.50	m	CH-3
	3.42	m	CH-4
	3.48	m	CH-5
	3.74, 3.91	m	CH_2-6
18. α-Glucose	**5.25**	**d**	**CH-1**
	3.55	m	CH-2
	3.72	m	CH-3
	3.42	m	CH-4
	3.84	m	CH-5
	3.73, 3.90	m	CH_2-6
19. Sucrose	**5.42**	**d**	**G CH-1**
	3.59	m	CH-2
	3.79	m	CH-3
	3.48	m	CH-4
	3.85	m	CH-5
	3.82	m	CH_2-6
	3.69	m	F CH_2-1′
	\	\	C-2
	4.22	m	CH-3′
	4.06	m	CH-4′
	3.90	m	CH-5′
	3.82	m	CH_2-6
20. 4-Hydroxycumarate	6.36	d	α-CH
	7.49	d	β-CH
	6.80	m	CH-2,6
	7.51	m	CH-3,5
21. Betalamic acid	**6.02**	**d**	**12-CH**
	6.32	bs	18-CH
	8.38	d	11-CH
22. Fumaric (FumA)	**6.51**	**s**	**CH=CH**
23. Dopamine	6.87	m	CH-2
	6.85	m	CH-4
	**6.73**	**pd**	**CH-5**
	3.22	t	α-CH_2
	2.88	t	β-CH_2
24. Betanin	7.04	bs	CH-4
	**7.11**	**bs**	**CH-7**

25. Tyr	**7.17**	**d**	**CH-2,6**
	6.89	d	CH-3,5
	3.15	dd	β-CH_2
	3.93	dd	α-CH
26. Phenylalanine (Phe)	7.32	d	CH-2,6
	7.38	d	CH-4
	**7.42**	**d**	**CH-3,5**
	3.27	m	β-CH_2
	3.98	dd	α-CH
27. Triptophan (Trp)	7.20	t	CH-5
	7.27	t	CH-6
	**7.53**	**d**	**CH-7**
	7.73	d	CH-4
28. 4-Hydroxybenzoate	7.80	d	CH-2,6
	6.84	d	CH-3,5
29. Fumaric acid (FA)	**8.46**	**s**	**CH**
30. Trigonelline	**9.11**	**s**	**CH-1**
	8.83	m	CH-3
	8.11	m	CH-4
	8.83	m	CH-5
	4.43	s	CH_3

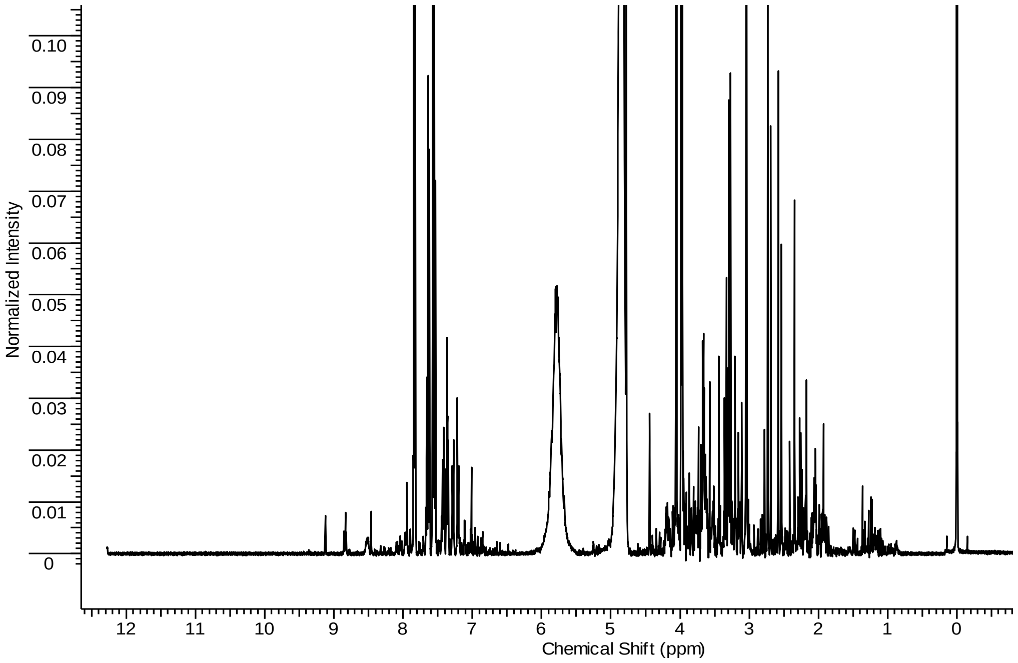

Figure S2. ^{1}H NMR urinary spectrum of a control sample (T0)

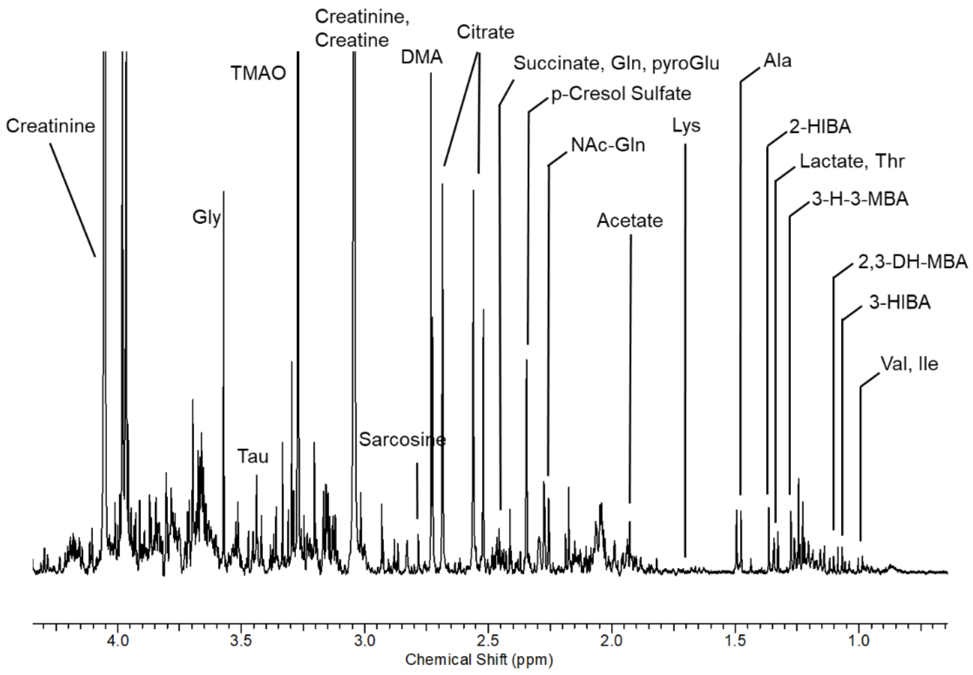

Figure S3. ^{1}H NMR spectrum of urine, from 1 ppm to 4 ppm.

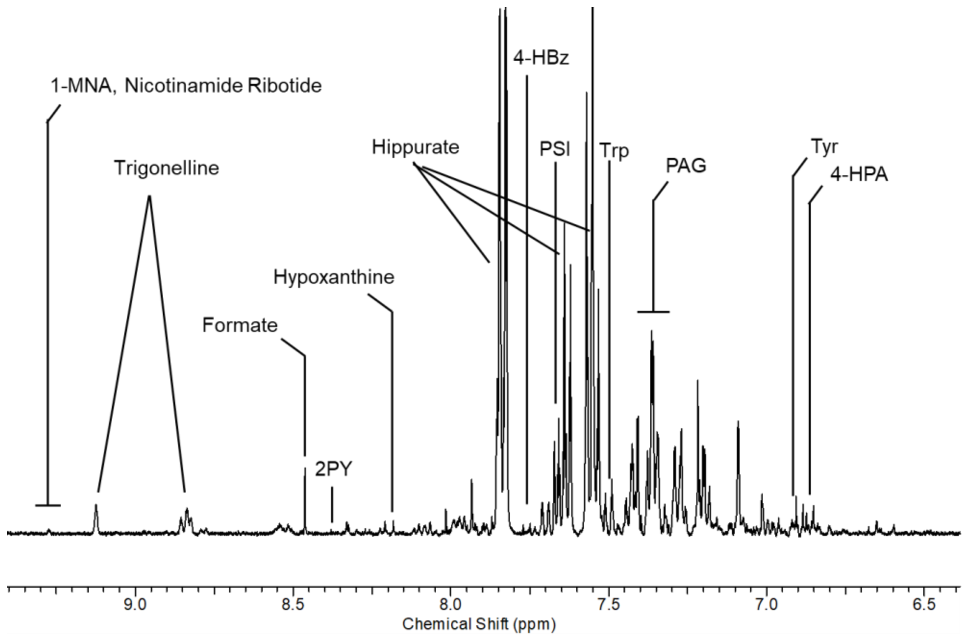

Figure S4. ^{1}H NMR spectrum of urine, from 6.5 ppm to 9 ppm.

Table S2. Resonance assignment of urine

Metabolite	^{1}H δ ppm	Multiplicity	Assignment
1. Isoleucine (Ile)	0.92<br>**1.01**<br>1.99	t<br>**d** 	CH_3<br>**CH_3**<br>CH
2. Valine (Val)	0.99<br>**1.05**	d<br>**d**	CH_3<br>**CH_3'**
3. 3-Hydroxyisobutyrate (3-HIB)	**1.07**<br>2.49<br>3.54<br>3.71	**d**	**CH_3**
4. Erythro-2,3-dihydroxybutyrate (Erythro-2,3-DHB)	**1.11**<br>4.19	**d**<br>m	**CH_3**<br>CH
5. 3-Hydroxy-3-methylbutyrate (3-H-3-MB)	**1.27**	**s**	**CH_3, CH_3'**
6. Lactate	**1.33**<br>4.11	**d**<br>q	**CH_3**<br>CH
7. Threonine (Thr)	**1.33**<br>3.59<br>4.26	**d**<br>d<br>m	**CH_3**<br>α-CH<br>β-CH
8. 2-Hydroxyisobutyrate (2-HIB)	**1.36**	**s**	**CH_3, CH_3'**
9. Alanine (Ala)	**1.49**<br>3.78	**d**<br>q	**CH_3**<br>α-CH
10. Acetate	**1.93**	**s**	**CH_3**
11. N-acetylglutamine (NAcGln)	1.95<br>2.12<br>**2.27**<br>4.18<br>7.97	 <br>**m**<br><br>bs	 <br>**CH_2**
12. Pyroglutamate (pyro-Glu)	2.03<br>**2.40**<br>2.50<br>4.19	m<br>**m**<br>m<br>dd	CH_2<br>**CH**<br>CH<br>CH
13. Glutamine (Gln)	2.13<br>**2.46**<br>3.78	m<br>**m**<br>t	CH_2<br>**CH_2**<br>CH
14. p-Cresol sulfate (p-CrS)	**2.35**<br>7.21<br>7.28	**bs**<br>dd<br>dd	**CH_3**<br>CH, CH′<br>CH, CH′
15. Citrate	**2.54**<br>2.69	**d**<br>d	**CH, CH′**<br>CH, CH′
16. Dimethylamine (DMA)	**2.73**	**s**	**CH_3, CH_3'**
17. Sarcosine (Sar)	**2.78**	**s**	**CH_3**
18. Dopamine-3-O-Sulfate (DA3S)	**2.94**<br>3.27<br>7.07<br>6.83<br>6.78	**t**<br>t<br>d<br>d<br>dd	**CH_2**<br>CH_2<br>CH-2<br>CH-5<br>CH-6
19. Creatine	**3.05**<br>3.95	**s**<br>s	**CH_3**<br>CH_2
20. Creatinine	3.03<br>**4.05**	s<br>**s**	CH_3<br>**CH_2**

21. Trimethylamine N-Oxide (TMAO)	**3.27**	**s**	**CH_3**<br>CH_2
22. Taurine (Tau)	3.27<br>**3.43**	t<br>**t**	CH_2<br>**CH_2**
23. Glycine (Gly)	**3.57**	**s**	**CH_2**
24. 4-Hydroxyphenylacetate (4-HPA)	**6.87**<br>7.17	**dd**<br>dd	**CH,CH**<br>CH,CH
25. Tyrosine (Tyr)	**6.90**<br>7.18	**dd**<br>dd	**2-CH,6-CH**<br>3-CH,5-CH
26. 4-Hydroxybenzoate (4-HBz)	6.97<br>**7.76**	dd<br>**dd**	2-CH,6-CH<br>**3-CH,5-CH**
27. Tryptophan (Trp)	7.20<br>7.27-<br>7.29-<br>**7.50**<br>7.70	-<br>-<br>-<br>**pd**<br>pd	3-CH<br>2-CH<br><br>**5-CH**<br>4-CH
28. Phenylacetylglycine (PAG)	**7.36**-7.42	**m**	**CH 2-5**
29. Hippurate (Hipp)	3.97<br>**7.55**<br>7.64<br>7.83	d<br>**m**<br>m<br>m	CH2<br>**3,5-CH**<br>4-CH<br>2,6-CH
30. Pseudouridine (PSI)	**7.67**	**bs**	**CH**
31. Hypoxanthine (Hyp)	**8.19**<br>8.21	**s**<br>s	**2-CH**<br>7-CH
32. N1-Methyl-2-pyridone-5-carboxamide (2PY)	3.64<br>6.67<br>7.98<br>**8.33**	s<br>d<br>d<br>**dd**	N-CH_3<br>3-CH<br>4-CH<br>**6-CH**
33. Formate	**8.46**	**s**	**CH**
34. 4-Methylpyridine-2-carboxylate (4MP2CA)	2.46<br>7.47<br>7.88<br>**8.48**	s<br>dd<br>**d**<br>**d**	CH3<br>CH-5<br>CH-3<br>**CH-6**
35. U01	8.06<br>8.54<br>**8.78**	 dd<br>**d**	 <br>**6-CH**
36. Trigonelline (Trig)	4.44<br>8.08<br>8.84<br>**9.12**	s<br>m<br>m<br>**s**	N-CH_3<br>5-CH<br>4,6-CH<br>**2-CH**
37. 1-Methylnicotinamide (1-MNA)	8.17<br>8.89<br>8.96<br>**9.28**	t<br>d<br>d<br>**s**	5-CH<br>4-CH<br>6-CH<br>**2-CH**
38. Nicotinamide Ribotide	**9.33**	**bs**	**6-CH**

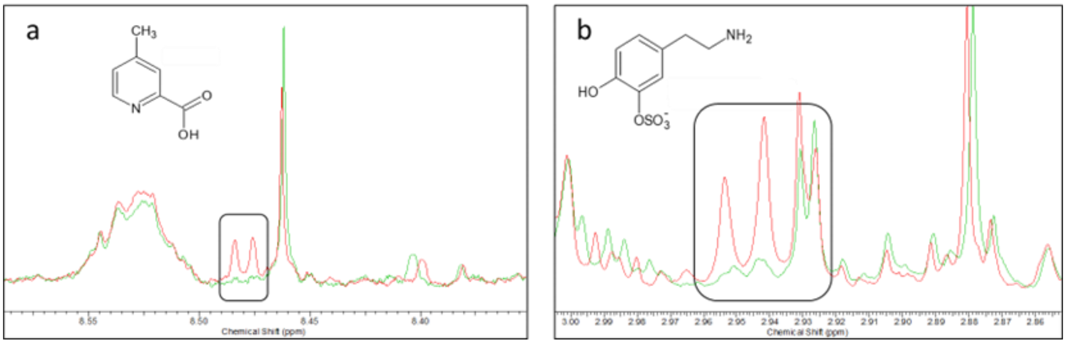

Supplementary Figure S5. ^{1}H NMR urine of one subject collected in day B (red) and day C (green) three hours after the juice intake (T3); a) 4-methylpyridine-2-carboxylic acid; b) dopamine-3-O-sulphate.

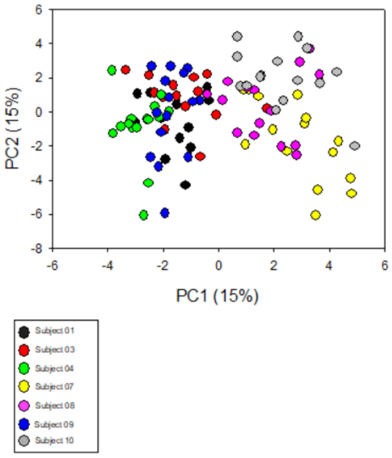

Figure S6. Scores plot of principal component analysis (PCA) performed on all the subjects, considering all five sampling times and all the experimental sessions.

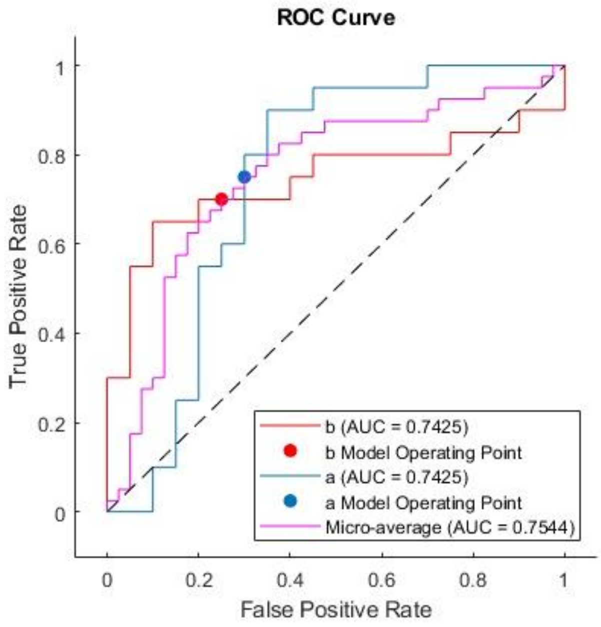

Figure S7. Area Under Receiver Operating Characteristics for PLS-DA between rest and physical activity being equal the RBJ intake (A vs B).

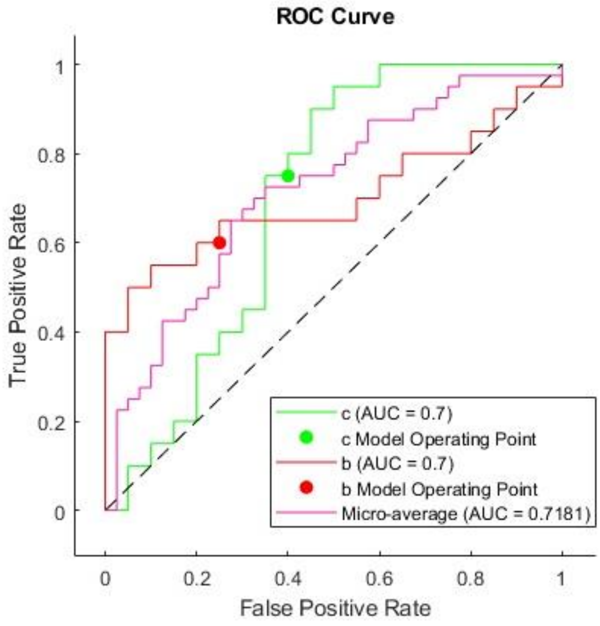

Figure S8. Area Under Receiver Operating Characteristics for PLS-DA between RBJ and placebo intake, being equal the physical activity (B vs C).

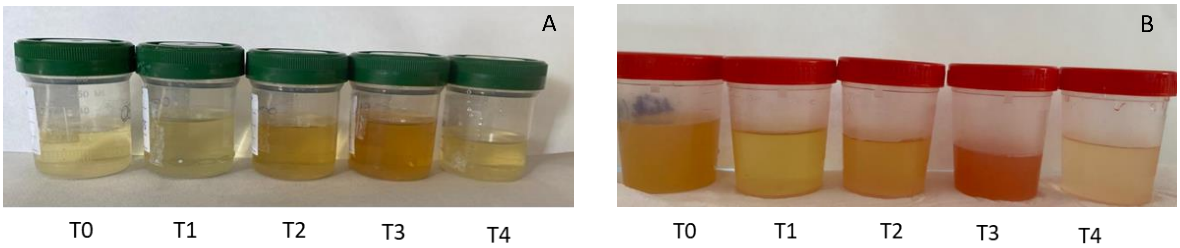

Figure S9. Picture of urine collected from the same subject during A) the experimental session with placebo and B) the experimental session with RBJ intake, being equal the physical activity.
